# Dialogue among Lymphocytes and Microglia in Glioblastoma Microenvironment

**DOI:** 10.3390/cancers14112632

**Published:** 2022-05-26

**Authors:** Alessandro Mormino, Stefano Garofalo

**Affiliations:** Department of Physiology and Pharmacology, Sapienza University, 00185 Rome, Italy; alessandro.mormino@uniroma1.it

**Keywords:** glioblastoma, microglia, cytotoxic lymphocytes, natural killer cells, adeno-associated viruses, CAR technology, enriched environment

## Abstract

**Simple Summary:**

In this review, we summarize in vitro and in vivo studies related to glioblastoma models and human patients to outline the symbiotic bidirectional interaction between microglia, lymphocytes, and tumor cells that develops during tumor progression. Particularly, we highlight the current experimental therapeutic approaches that aim to shape these interplays, such as adeno-associated virus (AAV) delivery and CAR-T and -NK cell infusion, and to modulate the tumor microenvironment in an anti-tumoral way, thus counteracting glioblastoma growth.

**Abstract:**

Microglia and lymphocytes are fundamental constituents of the glioblastoma microenvironment. In this review, we summarize the current state-of-the-art knowledge of the microglial role played in promoting the development and aggressive hallmarks of this deadly brain tumor. Particularly, we report in vitro and in vivo studies related to glioblastoma models and human patients to outline the symbiotic bidirectional interaction between microglia, lymphocytes, and tumor cells that develops during tumor progression. Furthermore, we highlight the current experimental therapeutic approaches that aim to shape these interplays, such as adeno-associated virus (AAV) delivery and CAR-T and -NK cell infusion, and to modulate the tumor microenvironment in an anti-tumoral way, thus counteracting glioblastoma growth.

## 1. Glioblastoma: The Most Aggressive Brain Tumor

Glioblastoma (GBM) is a high-grade glioma, representing the most common and lethal primary brain tumor in adults [1,2], characterized by high heterogeneity in terms of genetic and epigenetic modifications, histological hallmarks, and response to treatment [3,4]. GBM cells show a high ability to proliferate and invade the brain parenchyma, and the peculiar localization, shielded by the blood–brain barrier (BBB), enhances the resistance to several chemotherapeutic drugs [5], giving this tumor a poor prognosis despite the scientific progress and combination of surgery, chemotherapy, and radiotherapy [6,7,8]. The preferential routes of GBM cells to invade the parenchyma are along the blood vessels and white matter [9]. To efficiently migrate, GBM remodels the extracellular matrix through the expression of secreted proteases, including the MMP membrane types MMP1/14, MMP2, and MMP9, the serine protease uPA, and cell surface proteases of the ADAM family [9]. Interestingly, apart from the high invasion in the brain, GBM rarely forms metastasis out of the primary site, probably due to a particular tropism for brain areas [10,11,12,13,14,15,16].

Another pivotal hallmark of this brain tumor is the uncontrolled proliferation of GBM cells as a result of deregulation in several molecular pathways, such as p53 signaling. Loss-of-function mutations on the p53 protein have been found in many tumor malignancies, including GBM. The function of p53 can be deregulated by gain-of-function mutations of negative p53 regulators, such as MDM2 and MDM4, or loss-of-function mutations of its activators, such as p14ARF [17,18]. Furthermore, mutations in the PTEN protein, an inhibitor and part of the mTOR pathway, are epigenetically silenced or genetically mutated in almost 60% of GBM [19], and the deletion of chromosome 13, containing the gene Rb1 [7], results in uncontrolled tumor cell divisions [7,19]. Moreover, GBM supports its own growth by increasing angiogenesis, the ability to build up new blood vessels that are able to feed the tumor mass [20]. Recently, it was reported that angiogenesis is associated with the expression of the hypoxia inducible factor (HIF-1) in response to the hypoxia present in the tumor environment to produce pro-angiogenic factors such as vascular endothelial growth factor (VEGF) [21].

The genetic complexity shown in GBM involves several genetic and epigenetic modifications that result in the loss of tumor suppressor gene function (CDKN2A/B and PTEN) or the activation of oncogenic pathways (CDK4, p21–RAS, and MDM2) [22,23,24]. This genetic heterogeneity is accompanied by a high diversity in the cell populations forming the GBM microenvironment (GME), such as resident and peripheral immune cells, endothelial cells, mesenchymal cells, and glioma stem cells (GSCs) [25]. GSCs are characterized by the ability to differentiate into different cell lineages to reconstitute the tumor mass. This characteristic was first demonstrated with the identification of CD133^+^ GBM cells that were able to initiate the tumor process in vivo [26]. Furthermore, GSC cells show multiple drug resistance: it has been shown that CD133^+^ GBM cell fractions in the tumoral mass increase after exposure to radiation due to the activation of the DNA damage checkpoint response and an increase in DNA repair capacity [27]. Moreover, GBM cooperates with parenchymal cells in multiple ways: among them are soluble molecules [28,29,30,31,32,33,34,35,36], direct synaptic interactions [37,38,39], and extracellular vesicles [40], promoting tumor proliferation, angiogenesis, immunosuppression, degradation of the extracellular matrix, and invasion.

Improving the knowledge of the pathways driving the interactions between GBM, infiltrating cells, and microglial cells may provide new perspectives to manage GBM growth and development, highlighting the way for new therapeutical approaches and targets.

## 2. GBM–Microglia Symbiosis

In the last decades, our vision of tumor mass has been radically changed. Nowadays, it is well described that tumoral mass is not solely constituted by clonal cancer cells; indeed, there is great heterogeneity between the cancer cells inside the mass. Among them, glioma-associated resident microglia and peripheral-invading monocyte-derived macrophages (called GAMs) represent from 30% up to 50% of total cells in the tumor microenvironment [41,42,43,44], with several potentially overlapping functions [45]. Initially, during GBM development, the main population of GAMs cells is represented by microglia. Subsequently, with tumor development, there is a progressive increase in the number of infiltrating macrophages/monocytes, in response to molecular signals secreted by GBM, that weaken the BBB to recruit peripheral immune cells [46,47]. For a long time, the lack of specific markers has made the distinction between microglia and brain-infiltrating monocyte-derived macrophages difficult. Furthermore, the first experimental approaches used to deplete the bone marrow progenitors that induced damage to the BBB and consequent monocyte infiltration into the brain [48,49,50,51], such as whole-body irradiation, helped complicate this distinction. To date, this problem is partially overcome with head shielding during irradiation [52] and the enhancement of experimental techniques, such as RNA sequencing (RNA-seq), mapping studies, and single-cell RNA-seq [53,54,55,56], allowing us to investigate the different profiles inside the GAMs.

Once recruited, GAMs are educated by GBM cells toward an anti-inflammatory/pro-tumoral phenotype that releases a plethora of soluble molecules with pro-tumoral effects [57,58,59,60,61], sustaining the GME, the tumor growth [57,58,59], and angiogenesis [60]. Nevertheless, depletion experiments in mouse models have demonstrated that GAMs do not participate in gliomagenesis [62]. The CSF1-R (signaling pathway fundamental for microglia and macrophage survival) inhibition reduces GAMs’ recruitment in the tumor core, resulting in a reduction of tumor cell proliferation and invasion [63,64,65]. However, a CSF-1R inhibitor-based therapy failed to reach significant results in a phase II study for GBM patients [66].

The pattern of molecules produced by GBM cells, such as toll-like receptors (TLRs), GDNF, CXCRL1, and TGF-β, attracts and affects GAMs functions, supporting tumor growth [67,68,69,70,71,72,73]. Interestingly, the isocitrate dehydrogenase (IDH) mutation affects the production of these factors, resulting in different GMEs [29,30]. Indeed, non-mutated IDH1 supports an immunosuppressive ground through the activation of the Wnt/β-catenin pathway in GAMs, which shows different gene expression signatures with respect to IDH-mutant GBMs [74].

**Figure 1:**
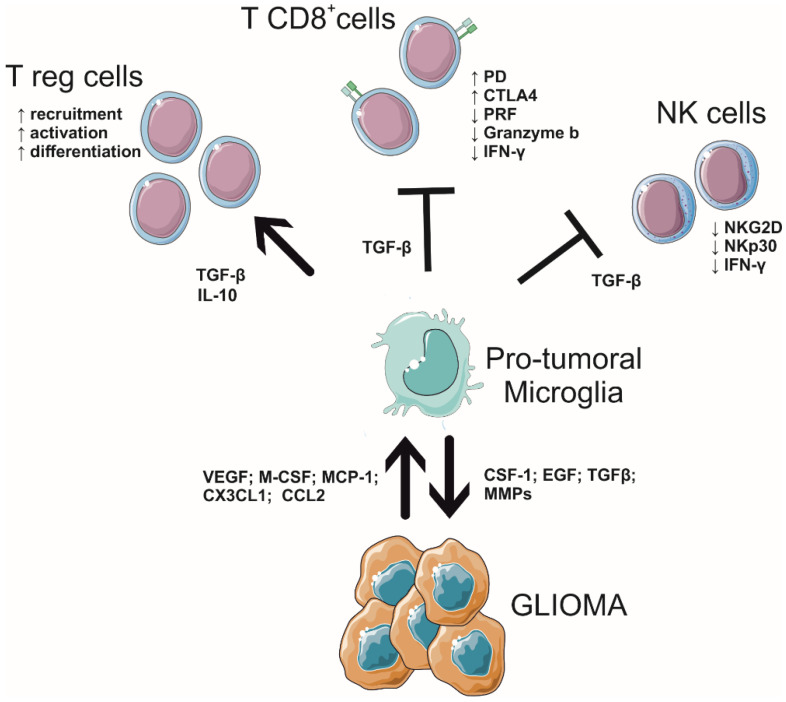
Scheme of interplay among microglia, lymphocytes, and glioblastoma in the tumor microenvironment.

The symbiotic interplay between GBM and GAMs (summarized in Figure 1), with the formation of an immunosuppressive microenvironment, makes this tumor resistant to chemotherapy and radiotherapy. For this reason, GAMs are becoming attractive for therapeutic research [75].

## 3. GAM Interactions with Lymphocytes in the GBM Microenvironment

A key element facilitating GBM growth is its ability to promote an immunosuppressive ground that hampers the reaction of immune cells against tumor cells. Consistently, GBMs show a weak infiltration of cytotoxic lymphocytes and a rare patient’s responsiveness to checkpoint inhibitor immunotherapy, classifying this brain tumor among the so-called “cold” cancers [4,76,77,78]. Indeed, the lymphocytic level positively correlates with increased survival in brain tumor patients, but no results have been produced in GBM patients [79,80,81,82]. Further, a comparative study of GBM patients exhibited lower T cytotoxic cell activity and higher Treg cell activation compared with healthy volunteers [83]. In this scenario, GAM interactions are not mainly restricted to dialogue with GBM cells; contrarily, GAMs orchestrate the immunosuppressive GME by communicating with brain parenchymal cells and infiltrated lymphocytes recruited by GBM and brain metastasis derived from extracranial cancers [79,80].

Both CD8^+^ and CD4^+^ T-lymphocytes, besides T helper, FoxP3^+^ Treg, myeloid suppressor cells, and natural killer (NK) cells, invade GBM [81,82,83,84,85,86,87]. Interestingly, mutated IDH 1 and 2 astrocytomas, which have a better prognosis in comparison with wild-type IDH, are related to a reduced number of cytotoxic lymphocytes in the tumor core [88,89]. To date, it is well described that GAM and T lymphocyte interactions drive GBM development, infiltration, and differentiation, and the complexity of this interplay forms the heterogeneity of GBM tissue across different patients. Particularly, the immunosuppressive ground created by GAMs, which inhibits the cytotoxic activity of T-cells and explains why GBM patients do not respond to immune therapy [90], is mainly due to the expression of PD ligands PD-L1/2, and the cytotoxic T lymphocyte-associated protein 4 (CTLA-4) ligands CD80 and CD86 [91]. Further, GAMs release TGF-β, a key signal that inhibits the anti-tumoral effects of T-cells [92], downregulating the expression of the proteins responsible for lymphocyte cytotoxicity, such as perforin, granzyme, and interferon (IFN)-γ; consistently, in vivo studies in GBM-bearing mice have shown that the neutralization of TGF-β upregulates the expression of these genes in CD8^+^ T-cells [92]. In contrast, GAM-originated TGF-β, with the support of the IL-10, stimulates the differentiation of naïve T-cells into regulatory T (Treg) cells, which suppress CD8^+^ T-cells in the GME [93,94]. Moreover, TGF-β induces downregulation of NKp30 and NKG2D activating receptors on NK cells [95]. These molecules also promote GBM angiogenesis, growth, and invasion and the reduction of T-cell cytotoxic activity [96,97,98,99,100,101].

GAMs are also able to regulate the infiltration of lymphocytes in malignant tumors. GAMs release the chemokine CCL2, which is essential for the recruitment of regulatory T-cells and myeloid-derived suppressor cells [102]. Furthermore, GAMs control extracellular matrix stiffness and collagen deposition, regulating the movement of T-cells across the GME [103]. Consistently, GAM depletion has been reported to increase CD8^+^ T-cell migration and infiltration [104], helping to overcome immunosuppression.

Among the patterns of GAM’s molecules released to create the immunosuppressive microenvironment for GBM, neuropilin-1 (NRP-1), expressed by various types of cells, including microglia and macrophages, plays a pivotal role [105,106]. NRP-1 increases angiogenesis (enhancing the production of pro-vascularization signals) and boosts the infiltration of Treg in the tumor mass while decreasing the number of T CD8^+^ lymphocytes and driving GAM polarization toward a pro-tumoral way [107,108,109]. Consistently, the depletion of NRP-1 from microglia in glioma-bearing mice leads to a reduction in GBM volume, increasing the number of T CD8^+^ cells in the tumor mass and shaping GAM polarization [109].

The GAM’s role in driving immune responses against the GBM makes these cells a juicy target for several experimental immunotherapeutic studies that aim to reprogram microglia or macrophages to counteract tumor development.

### 3.1. Activating Lymphocytes to Modulate Microglia-GBM Cross-Talk

Given the evidence on the tumoricidal role of microglial cells when they are activated toward a pro-inflammatory phenotype, in the last years, one big effort of biomedical research has aimed to re-educate microglia against tumor cells. Consistently targeting the immune tumor microenvironment appears to be a promising therapeutic strategy to counteract GBM progression [5,33,110]. The switch to a specific phenotype correlates with prognosis, and the pathological assessment of a specific microglial setting can predict a patient’s outcome [111]. Microglia polarization is mediated by complex pathways involving cross-talk with GBM and immune cells. In this scenario, both environmental and peripheral stimuli seem to play a central role. In particular, recently, evidence has demonstrated that activated lymphocytes can modulate GAM phenotypes, highlighting a new potential target able to drive microglia against GBM.

#### 3.1.1. Engineered Microglia Boost Lymphocyte Functions against GBM

Gene therapies for GBM are being developed in clinical trials; particularly, in recent years, more and more studies have aimed to genetically manipulate microglial cells as a new effective therapeutical approach to defeating several neurodegenerative diseases [112,113,114]. The use of recombinant viruses such as adeno-associated viruses (AAVs), a small and non-pathogenic defective parvovirus, is a promising tool due to their characteristics, such as high titers, broad host range, efficient infection of quiescent cells, and vector integration [115]. In this way, AAVs represent an efficient vector system, determining long-lasting changes in gene expression even if the limited gene transfer to GBM cells hampers its use [116]. Consistently, AAVs are considered safe for human gene therapy and have been successfully used to target several cell types within the central (CNS) and peripheral (PNS) nervous systems, including neurons, oligodendrocytes, astrocytes, Müller glia, and Schwann cells [117]. On the other side, the AAV transduction of microglia is rare and challenging: indeed, in vivo, less than 20% of efficiency seems to be achieved, although some cases of microglial transductions, both in vitro and in vivo, have recently been reported, thanks to advances in the new strategies designed for recombinant viral vectors [113,118,119]. Furthermore, engineered microglial cells could be destined as a biologically active vehicle for the delivery of anti-tumoral molecules. Indeed, recently, the potential use of microglia engineered to express IL-15 upon infection with a recombinant AAV serotype 2 (rAAV2) carrying IL-15 (rAAV2-IL-15) was explored to counteract GBM growth in mouse models [119]. IL-15 is a crucial cytokine for the development, maturation, and activation of NK cells and CD8^+^ T-cells, with no effect on the expansion of the T regulatory cell population involved in suppressing immune responses, highlighting a potential therapeutic use in cancer immunotherapy [120]. Furthermore, IL-15 enhances the anti-tumor efficacy of the extracellular vesicles derived from NK cells, showing higher cytolytic activity against GBM [121]. Microglia infected with rAAV2-IL-15 functionally induce the release of IL-15, increasing the viability of NK cells without affecting their activation state in vitro [119]. In vivo, the rAAV2-IL-15 microglial cells infiltrate GBM mass and increase the recruitment of IFN-γ^+^ NK cells in GBM-bearing mice, with effects on tumor growth [32,33,119], highlighting the fundamental role of IL-15 in the tumor core to boost immune reaction. Moreover, rAAV2-IL-15 microglia consistently modulate the GAM state, with a reduction in arginase levels and an increased number of branches, and cover the parenchymal region [119], suggesting the switch to an anti-tumoral phenotype [122]. These data indicate that the recruited NK cells in the tumor core are activated and release pro-inflammatory cytokines (i.e., IFN-γ), thus explaining the modulation of the GAM phenotype [33].

An elegant approach to modulating microglial functions in GBM using AAV delivery is the intracranial injection of rAAV2 that encodes IL-12 in rat models. rAAV2-IL-12 increases the expression of IL-12 and IFN-γ in the brain, potent cytokines that enhance microglial activity [118,123,124]. Consistently, the use of rAAV2-IL-12 increases microglial infiltration in GBM and the expression of the activation markers ED1 and tumor necrosis factor-related apoptosis-inducing ligand (TRAIL), turning in the acquisition of an anti-tumoral phenotype by microglia, which is associated with a reduction in tumor volume and longer survival time in rat models [118].

**Figure 2:**
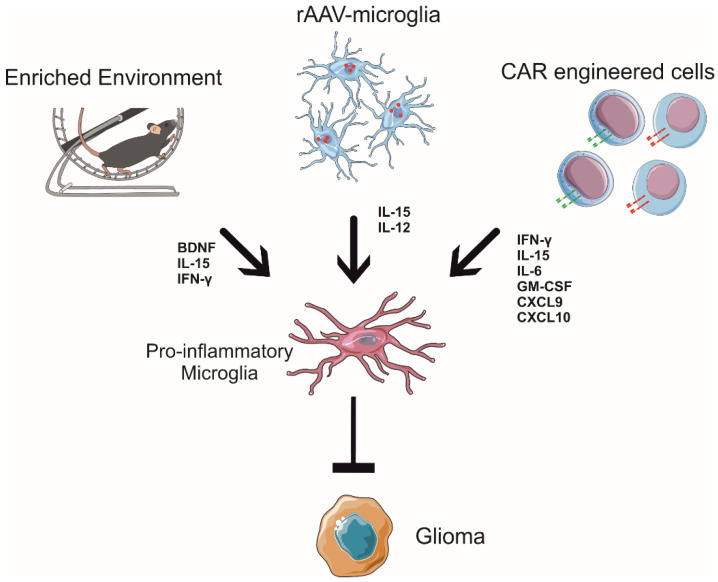
Environmental stimuli, engineered microglia, and CAR-lymphocytes shape the glioblastoma microenvironment, educating microglial functions in an anti-tumoral way.

These data demonstrate the potential for improved AAV-based gene therapy for GBM-targeting microglial cells as a vehicle and tool to translate the anti-tumoral signals inside the tumor mass, boosting the lymphocytes’ tumoricidal activity and offering a new perspective to use them as Trojan horses to modify the tumor microenvironment (Figure 2).

#### 3.1.2. Environmental Stimuli Boost the Interplay between Lymphocytes and Microglia, Reducing GBM Growth

Lifestyle, which includes many aspects of interactions with the environment, from nourishment and education to physical activity and quality of sleep, is one of the most powerful instruments shaping mankind. Exposure to different environments affects brain functions and cognitive performance [125,126,127]. Clinical studies have demonstrated that depression, feelings of loneliness, and low sociability represent important risks for the development of several types of cancers [128]. On the contrary, in humans, positive stimuli such as motor activity, social interaction, and cognitive stimulation related to, for example, art or music can boost neuronal connectivity and counteract cancer development [125,126,127,128], supporting the idea that patients should benefit from an improved lifestyle.

In mouse models, the enriched environment refers to housing animals in larger cages with various possibilities of physical activities and exploration, using objectives such as ladders, running wheels, plastic tubes, and other toys [129]. Enriched environment exposure has beneficial effects on several neuronal activities in mice, improving spatial memory, increasing dendritic arborization and the density of dendritic spines on cortical neurons [130], and exhibiting higher hippocampal neurogenesis in adults [131]. In particular, physical exercise, exposure to an enriched environment, and dieting act through complex modifications of microglial cells, which change their phenotype and modulate their functional activity [132]. All these environmental stimuli are able to be converted into molecular signals in the brain that educate microglial cells to remodel brain homeostasis and shape neural plasticity, enhancing neuroprotection and counteracting the development of several pathologies [133,134,135]. Among the potential candidates for this communication, brain-derived neurotrophic factor (BDNF) and nerve growth factor (NGF) are key cerebral mediators of these phenomena [136,137,138,139,140]. With regard to cancer, clinical studies demonstrate that specific distressing stimuli, such as depression, feelings of loneliness, and lack of social relationships, represent important risk factors for tumor development and progression [128]. In contrast, it is known that living in environments that are enriched with sensorial, physical, and social stimuli affects the levels of hormones linked to the hypothalamic–pituitary axis, such as norepinephrine, BDNF, and glucocorticoids, regulating the growth of several types of tumors in both humans and mouse models [141,142,143]. In GBM, environmental stimulation shapes microglial toward an anti-tumoral profile [32,33]. Indeed, housing animals in an enriched environment deeply modifies GAM phenotypes, in particular the microglial phenotype, as shown by gene expression profiling: myeloid cells, isolated from the brain of glioma-bearing mice, show a reduction in genes related to the pro-tumoral phenotype, but only microglia increase the expression of pro-inflammatory genes, indicating an anti-tumoral state [33,58]. Furthermore, environmental stimuli modify the morphology of GAMs infiltrated in tumor mass, reducing cell body roundness and increasing the length and number of cell branches, the speed of process movement towards ATP (which mimics an injury signal), and the expression of P2ry12 mRNA, thus suggesting the re-establishment of a more efficient homeostatic and patrolling activity of these cells [144]. Interestingly, P2RY12 is specifically expressed by microglia and is associated with ATP-dependent process patrolling [145] and better survival of patients with astrocytoma [146]. Lastly, exposure to environmental enrichment modulates microglia phagocytic activity [33]. This effect of the environment on microglial cells in GBM is mediated by the NK cells. In fact, during enriched environment exposure, the NK cells more efficiently colonize the brain, producing IFN-γ and degranulating against GBM cells [32]. Moreover, upon housing in an enriched environment, there was a significant increase in direct contact between GBM and NK cells, and the NK cell depletion completely abolished the effect of the environment on pro- and anti-inflammatory gene expression in GAMs [33]. The interplay between microglia and NK cells was, at least in part, orchestrated by the IFN-γ released by the recruited NK cells and the IL-15 released by microglial cells upon environmental stimuli exposure [33]. Consistently, the BDNF produced in the brain of glioma-bearing mice after enriched environment exposure stimulates the production of IL-15 by microglial cells, which, in turn, stimulates NK cells to produce IFN-γ, with effects on GAM phenotypes, switching them towards an anti-tumoral state (see Figure 2), which explains the protective effects of the environment.

#### 3.1.3. CAR Technology in GBM

Recently, chimeric antigen receptor (CAR) technology has been shown to be a valid approach to counteract the growth of several types of cancers [147,148]. This technology takes advantage of direct immune cells, particularly T-lymphocytes, against tumors. In detail, isolated T-lymphocytes from patients are engineered to express a chimeric receptor directed against tumoral antigens. Once generated, these CAR-T cells are expanded in vitro and, subsequently, are re-infused into the donor patient [149]. One of the main advantages of CAR technology is that the chimeric receptor has a higher affinity to its target compared to the T-cell receptor (TCR) expressed on the membrane of lymphocytes [150]. Furthermore, the binding of the receptor with the antigen is not mediated by major histocompatibility complexes (MHCs); in this way, CAR-T cells are insensitive to the loss of MHCs used by tumors as an immunoescaping strategy [150]. Indeed, CAR-T cell-based therapy showed great clinical success in fighting hematological malignancies [151,152,153,154], and several clinical trials were conducted exploring the use of CAR-T cells against solid tumors, including GBM [155,156,157,158].

One of the initial targets of CAR-T cells in GBM therapy was IL-13 receptor IL13Rα2, which has been found to be overexpressed in human GBM samples [159]. The first generation of IL13Rα2 CAR-T cells is able to discriminate GBM cells from normal cells and selectively exert cytolytic activity in vitro and in GBM-bearing mice [160]. A second generation of IL13Rα2 CAR-T cells was generated to overcome the problem of persistence and to enhance biological activity in terms of cytotoxicity and pro-inflammatory cytokine production [161]. After the infusions, increased levels of pro-inflammatory cytokines were detected in cerebrospinal fluid (CSF), including IFN-γ, IL-15, IL-6, IL-10, GM-CSF, IL-2, IL-2Ra, IL-1RA, CXCL10, granzyme b [162], and, interestingly, the chemokines CXCL9 and CXCL10 [163]. These ligands for the CXCR3 receptor are expressed by macrophages and microglia and could modulate the activation state of GAMs [164]; consistently, CXCR3-lacking macrophages promote cancer growth [164]. Moreover, CXCL9 and CXCL10 regulate the recruitment of T and NK cells in GBM [165].

Another CAR-T cell target is mutated epidermal growth factor receptor variant III (EGFRvIII), overexpressed in a subset of GBM patients [166]. These engineered EGFRvIII CAR-T cells selectively recognize and kill GBM cell lines in vitro [166] and produce pro-inflammatory cytokines [166,167,168] that are able to increase the survival of human and mouse models [167]. Although these are promising results, EGFRvIII CAR-T cells must overcome the problem of the heterogeneous expression of the receptor in GBM samples, the secondary effects on other cells expressing the EGF receptor, and the increased expression of immunocheckpoints by tumor cells [168]. Recently, CAR technology investigated the possibility of targeting epidermal growth factor receptor 2 (HER2) and the integrin protein α_V_β_3_, with promising results. These proteins are highly expressed by many solid tumors, including GBM and diffuse intrinsic pontine glioma (DIPG), while it is minimally expressed in physiological tissues [169,170]. In a preclinical study, HER2 and α_V_β_3_ CAR-T cells induced death in human and murine GBM cells and, in CD133^+^ GSCs, increased the production of pro-inflammatory cytokines, such as IFN-γ, TNF-α, and IL-2, in co-culture experiments in vitro. Furthermore, both HER2 and α_V_β_3_ CAR-T cells significantly prolonged the survival of GBM xenograft mice, reducing tumor growth [170,171]. Although these are promising results, more studies are needed regarding their safety and efficacy in human GBM patients [149]. Moreover, α_V_β_3_ CAR-T cells have been shown to develop memory and persist for a long term in mouse models [170]. The beneficial effects on the tumor mass can also be ascribed to re-educate GAMs toward an anti-tumoral phenotype.

All the CAR-T cells examined in this review showed increased production of IFN-γ and other pro-inflammatory cytokines. It has been demonstrated that IFN-γ can polarize microglia toward a pro-inflammatory phenotype with the upregulation of pro-inflammatory genes such as IL-1β, IL-6, TNF-α, NOS2, and CD86 [172]. The release of IFN-γ by CAR-T cells in the tumor mass can also affect the GAM population in the GME in a pro-inflammatory way.

With regard to NK cells, it is also interesting that these lymphocytes have been engineered using CAR technology to be efficient tools against GBM. Han and collaborators, in 2017, demonstrated that NK cell-targeting non-mutated EGFR and mutated EGFRvIII showed enhanced anti-tumor activity and increased production of IFN-γ in vitro. Furthermore, the intracranial administration of CAR-NK cells led to reduced tumor growth and increased glioma-bearing mice survival [173]. Furthermore, another CAR-NK cell target is ErbB2/HER2. These cells exhibit high cytotoxic activity on ErbB2^+^ GBM cells, in both in vitro and in vivo models. Moreover, immunocompetent mice showed resistance to tumor growth and development when re-challenged with successive GBM infusions, proof of the induction of long-lasting immunological memory [122]. ErbB2 CAR-NK cells actively produce IFN-γ, TNF-α, IL-10, and the chemokine macrophage inflammatory protein MIP-1α when co-cultured with ErbB2^+^ cells [174], possibly modulating microglial behavior [33].

These findings suggest that the use of CAR-T cells and CAR-NK cells has beneficial effects because of the direct cytotoxic activity on tumor cells and through the creation of an inflammatory microenvironment that can revert GAM phenotypes and behavior toward the anti-tumoral phenotype.

## 4. Conclusions

GBM represents 81% of primary brain tumors [175]. Despite the recent and accurate classification of all gliomas and the scientific findings regarding molecular mechanisms at the base of their properties, the GBM remains a devastating tumor. Furthermore, in GBM, recurrence is inevitable; the current improvement in surgery, chemotherapy, or radiotherapy increases the mean survival rate of GBM patients by only a few months, mainly due to treatment resistance and a lack of response to targeted therapies. The resistance to the therapies is due to GBM heterogeneity, hypermutation, and oncologically activated alternative molecular pathways that shape the tumor microenvironment to facilitate therapy failure [176]. Moreover, GBM promotes an immunosuppressive microenvironment, supported by infiltrated macrophages and brain resident microglia, that hampers an effective immune reaction against glioma cells, promoting immunotherapy failure [57,58,59]. In this scenario, microglial cells have dialogues with infiltrated lymphocytes, and these interactions play key roles in GBM progression.

Here, we review the state-of-the-art regarding this fascinating cellular communication, highlighting the current hypothesis that modulating this interaction could represent a promising therapeutical approach. The first approach is to engineer microglia using AAV delivery, with the aim of modifying the expression profiles of these cells in order to induce a pro-inflammatory microenvironment, contrasting tumor growth and recruiting competent immune cells that are able to exert cytotoxic activity [118,119]. The second strategy is to exploit environmental stimuli to re-educate microglia and infiltrated lymphocytes in an anti-tumoral interplay, with the release of cytokines that reinforce pro-inflammatory ground, thus creating a virtuous circle [33,143]. The last examined strategy is the direct engineering of T-lymphocytes and NK cells with CAR technology. The purpose of this method is to create personalized therapy that is selectively directed against GBM antigens [161,168,169,170].

In conclusion, it is crucial to keep improving the biological knowledge of GBM and the interplay with resident and infiltrating immune cells in order to understand cell-to-cell communication mechanisms and their role in driving tumor pathogenesis. The possibility of integrating these exciting discoveries with new combination therapies will open new tools for treating this devastating disease.
